# Integrated challenges for IMGs as they migrate and move into general practice careers in rural Australia: a multi-staged qualitative study

**DOI:** 10.1186/s12909-026-08657-2

**Published:** 2026-01-31

**Authors:** Belinda O’Sullivan, Kim Omond, Neysan Sedaghat

**Affiliations:** 1 School of Rural Health, Monash University, 26 Mercy St, Bendigo, Victoria 3550 Australia; 2 General Practice Supervision Australia (GPSA), Level 40/140 William Street Melbourne, Melbourne, Victoria 3000 Australia

**Keywords:** International medical graduates (IMGs), Rural general practice, Medical education, Supervision, Satisfaction, Retention

## Abstract

**Background:**

International medical graduates (IMGs) are important to supplement domestic workforce gaps in rural general practice in Australia. However, there is no evidence about how to support IMGs longitudinally to achieve such careers. This research aimed to explore the integrated challenges for IMGs across the pathway from migrating to specialising as a general practitioner (GP) in a rural location, to inform what coordinated strategies might be needed.

**Methods:**

A qualitative participatory action research study of multi-staged one-hour semi-structured qualitative interviews and two-hour focus groups exploring IMG experiences from migration to pursuing rural GP careers in Australia. Participants had different roles across the prevocational and vocational rural general practice training system; IMGs were prioritised. An initial focus group in February 2025, consulted a 10-person Project Advisory Group. Data were transcribed and deductively and inductively coded for themes. Between March and May 2025 further focus groups and interviews were done with 31 wider participants, to deepen thematic insights. In July 2025, the Project Advisory Group we re-consulted to refine and confirm the themes.

**Results:**

Overall, 68% of respondents were IMGs; 46% from towns < 15,000 population and a range of roles across rural general practice training. We found three temporal stages representing different challenges: migrating and acclimatising; moving to new workplaces and communities; and training as a specialist GP in a rural location. Cross-cutting themes were complexity and frustration and the informal exchange of information and advice from other IMGs over these stages. Sub-themes identified specific issues at each stage including, limited access to relevant and tailored information for planning their life and career, limited GP career mentorship and tailored training advice and insecure pathways with variable quality supervised learning. IMGs also lacked recognition of past training and experience and had limited opportunities to address skills gaps through nuanced learning to help them adjust to the scope and responsibilities involved in rural GP careers.

**Conclusions:**

IMGs may experience three-staged challenges and specific issues as they navigate the pathway to establishing a rural GP career in Australia. To mitigate an overreliance on informal supports between IMGs, targeted and coordinated resources, education and training may be needed.

## Introduction

Most high-income countries use migrant doctors to supplement areas of workforce shortages, with the expectation that the host country will equitably support their careers [1]. Australia is a useful case study of a country which uses international medical graduates (IMGs) to buffer its rural general practice workforce [2–4]. IMGs are encouraged to work rurally through a moratorium (since the 1990s) and wider market pressures [5]. They constitute around two thirds of general practitioners (GPs) in small rural communities < 15,000 population, which has been growing despite introducing multiple strategies to promote GPs through domestic pathways [3, 4, 6]. A recent independent review of regulatory policies related to overseas health workers in Australia in 2023, advocates for greater use of IMGs in response to Australia’s shortfall of GPs, growing to 6,100 full time equivalent by 2048 [7, 8]. However, beyond a moratorium which Australia places on IMGs to direct them towards areas of workforce shortages, there is no national strategy to support IMGs into rural GP careers [5]. As part of a larger project to develop a roadmap of strategies to support IMGs on the pathway into GP careers in Australia, this research aimed to understand the integrated challenges of IMGs across the journey from migration to becoming a GP specialist in rural Australia.

There is a wide literature about IMG challenges although most is focused on hospitals and metropolitan locations, limited to one stage of assessment, employment, training program or issue, rather than taking a longitudinal focus [9–11]. Attending to the rural GP context is important because rural GP careers involve issues like relocating, living in smaller communities with distinct cultural norms, and working more autonomously across a diverse and unpredictable caseload [12, 13]. Australia’s rural context (Box 1) involves large distances and distributed towns, which can add to professional isolation. Interview-based research found that unsupported IMGs encountering high workloads in rural general practice in Australia ended up pursuing a more limited scope of work [14].

**Table:** 

Box 1: Australian rural context and general practice
Australia is vast, spanning more than 7.7 million square kilometres and encompassing a wide range of geographical diversity [15].
The population exceeds 26 million with approximately 28% living outside the major cities, spread across more than 12,300 rural localities covering 99% of Australia’s land area [16].
Access to rural general practitioners (GPs) is under pressure due to the rising wave of medical workforce non-GP specialisation among domestic graduates with limited uptake of rural careers despite strong investment in programs providing rural immersion for medical students [17, 18].

Consolidating an understanding of the IMG challenges on the pathway to rural GP careers in Australia is important because policy action is easily stymied by the complexity of the IMG journey. The standard (registration) pathway, through which most IMGs enter Australia, requires engagement with multiple agencies, is a useful barometer of the average experience (summarised in Box 2). However, for most IMGs, there are many touchpoints with different siloed agencies, requisite paperwork, assessments and conditions around working and training to adhere to. A 2024 survey report by the Australian Medical Council (the national body responsible for assessing the entry-level skills and knowledge of IMGs), described that IMGs commonly encounter family pressures, systems issues and work and skills-related challenges [19]. Other literature reviews, not limited to Australia, notes that IMGs may face challenges clearing formal requirements and adjusting to cultural, educational and professional differences, impacting their identity and belonging [20–22]. Qualitative research about IMGs training in various Canadian specialties suggested IMGs feel disorientated and need time to adapt, before achieving integration into professions in a new country [23].

Specific to the GP training period, research from the United Kingdom noted IMGs require enhanced induction, personalised learning and exam planning to progress in training [24]. A qualitative study with Australia’s GP training stakeholders added that modularised learning under supervision with direct observation can also help [9]. Another study exploring a mostly IMGs cohort of GP trainees in more remote areas identified that they not only need support for competency building, but require strategies to facilitate confidence, competence, belonging and bonding [25]. However, there is no published material showcasing an integrated view of the challenges over the longitudinal pathway (from migration to pre-vocational and vocational steps) to achieve the desired career endpoint of rural general practice, which is what motivated this research.

**Table:** 

Box 2: Context for IMGs pursuing entering Australia and pursuing general practice careers
To work as a general practitioner (GP) in Australia (primary healthcare physician), IMGs complete a multi-step process involving assessment, registration, employment and entry into vocational training. Entry-level skills and knowledge is assessed for IMGs to go down different pathways depending on background skills and experience [26]. An estimated 60,000 IMGs interacted with the Australian Medical Council (AMC) from 2012 to 2023 [19]. Most IMGs take the standard pathway (no speciality GP qualifications from comparable countries which would allow assessment under other pathways) [26]. Such applicants are mostly from India, Pakistan, Bangladesh, Sri Lanka, China, Iran, Philippines, Myanmar, Russia and Iraq [19].
Firstly, English proficiency and previous qualifications are verified. IMGs then need to pass AMC examinations. The first is a multiple-choice questionnaire exam, often referred to as AMC part 1 which has a 50% pass rate [19]. After passing, some IMGs may be eligible for limited registration where they can practice under supervision in a hospital or general practice. To work in general practice, IMGs must find a role in an area of workforce shortage, typically rural or remote locations classified Modified Monash Model (MMM) 2-7, for around ten years, to access a billing provider number, due to a rural moratorium [5, 27].
Whether working already or seeking work, a second examination is required if IMGs seek provisional registration. This is either a clinical skills examination, the AMC part 2, which has a 35% pass rate or a workplace-based assessment (available in limited sites) with a 99% pass rate [19]. After the part 2 exam is completed, IMGs may apply for provisional registration through Australian Health Practitioner Regulation Agency (AHPRA). Under provisional registration, IMGs need to work under supervision and gain experience required for eligibility for general registration.
IMGs generally find their own employment to match their registration level. They may use private recruitment agencies or rural workforce agencies for assistance. Regardless of their registration, they must complete a pre-employment structured clinical interview for eligibility to work at the scope required in the specific general practice where they are seeking employment. IMGs may also choose to apply for and enrol in bridging programs (short-term, limited places mostly in state-based hospitals, subject meeting the eligibility criteria and being competitively selected) and/or use private resources at personal cost.
IMGs are typically eligible for GP specialty training after completing 12 months of supervised experience (in hospital or the community). Specialty GP training currently encompasses around six different programs through the option of two different colleges the Royal Australian College of General Practitioners and Australian College of Rural and Remote Medicine , each with different eligibility criteria around registration (limited to general), previous experience and work location and supervision requirements [28].
To assist IMGs and other prevocational doctors to access roles and prepare to enter specialty training as a GP in a rural location, the Australian government funds a Pre-Fellowship Program at the prevocational level [29]. This involves learning plans and assessments and expectations that candidates will apply for formal GP specialty training.

## Methods

This study involved multi-staged semi-structured interviews and focus groups based on a qualitative constructivist epistemology [30, 31]. All participants were informed that the results would be applied to a larger project to develop a “whole of pathway” roadmap to support IMGs into rural GP careers. Recognising the importance of engaging across a diverse and distributed rural GP training sector and any hard-to-reach groups like IMGs and rural participants, we also drew from participatory action research methods [32]. We included a recent IMG GP fellow within the research team (NS) and engaged a 10-person Project Advisory Group of mostly IMGs with different roles across the prevocational and vocational rural GP training system. The Project Advisory Group worked with the research team to inform the study aim and design, contribute as end users potentially affected by the research and to help to refine and confirm the results, as further described.

### Procedure

The Project Advisory Group and key contacts in agencies involved in the IMG pathway (known to the research team), were contacted by email and asked to circulate an invitation to the study via their responsible executive or manager. They were encouraged to snowball the invitation to widen the sampling frame. The enrolment process sought an expression of interest from IMGs and rural GP trainees, supervisors of IMGs and key informants across different prevocational and vocational rural GP training pathways in Australia. After reading the study information, (a process that the Project Advisory Group also completed) interested participants completed an expression of interest (Google Form), collecting basic information and written informed consent to participate in relevant phases of the research (Project Advisory Group consented to phases 1 and 2 and other participants consented to phase 2). When enrolling, respondents had the option to nominate for individual interviews rather than focus groups, and to bring a support person for their own comfort. In the study information, the participants were informed who the researchers were, their independence regarding collecting and interpreting the data, the purpose of the study and that places in the study were capped due to time and funding.

The Project Advisory Group were already pre-determined, however, for phase 2 participants, researchers reviewed the expression of interest data and purposefully sampled for IMGs and others across different rural GP pathways and roles including participants who were decision-makers, involved in training delivery, supervising IMGs and IMG trainees. This aligned with maximum variation sampling, with the aim of garnering a whole-of-sector perspective [33]. The data were collected in three stages (Table 1) to allow the research team to gradually build, deepen and confirm the concepts and themes.

**Table 1:** Phases of data collection

Stage	Who, what	Purpose
Phase 1 February 2025	Initial Project Advisory Group focus group consultation	To gather broad perspectives as end users of the research
Phase 2 March-May 2025	Semi-structured interviews and focus groups with wider participants	To broaden perspectives, building on the results from phase 1
Phase 3 July 2025	Final Project Advisory Group focus group	To refine and confirm a summary of the final themes

For each phase of data collection, participants were emailed to organise interviews and focus groups relative to their availability and preferences. Interviews were also offered where there might be unequal power between participants, such as supervisors and trainees, to promote psychological safety for IMG disclosure and for the convenience of scheduling around busy participants. The question frame was developed based upon the background research (Box 3) [9, 20, 21, 24, 34].

**Table:** 

Box 3: The question frame
Phases 1 and 2 - Tell me a bit about:
• Your background and experience related to IMGs in Australia?
• The IMG experience related to migration and settling into life and work in Australia?
• The IMG experience related to moving to new workplaces and rural communities?
• The IMG experience with navigating rural general practice training?
• What strategies could help IMGs at these stages?
• Is there anything else you would like to add?
Phase 3
• What do think about the findings?
• Are there any particular strengths or weaknesses?
• Do you have anything to add to the findings or their interpretation?
• Could anything be clarified?

Questions were informally piloted by the research team and circulated a week in advance of focus groups and interviews, to promote reflection and comfort. Participants were offered a $125 gift voucher/hour in recognition of their time.

### Data collection

An experienced PhD-trained rural workforce qualitative researcher (BOS) and GP academic fellow and medical educator (KO) (both women, motivated to understand the challenges so as to inform a roadmap of strategies), led the data collection using online recorded Zoom focus groups and interviews in phases 1–3. BOS was involved in leading most of the data collection to promote consistency in the style and technique. At the beginning of each consultation, participants were briefed about the background and aims and asked the specific questions for the phase of the research (questions displayed during the consultations using PowerPoint and screen sharing via Zoom).

Selected participants were known to the researchers, but all were prompted for honest and critical insights. Prompts like ‘tell me more’, why’, ‘for whom’, ‘when’ and ‘how’, were used to expand insights. For the focus groups, participants were encouraged to talk openly, and to comment on each other’s feedback to promote richer insights and all participants were encouraged to contribute [35].

All data were transcribed using Turboscribe, de-identified and assigned a unique identification. The transcripts and reflective notes from the researchers were used to discuss the emerging findings at weekly meetings and as a block, between each phase. Through this process the researchers considered any further lines of questioning needed and confirmed the questions for the next phase of the data collection. The overall question topics did not change but prompting expanded over time to explore new insights or gaps in the data. By around the 35th participant (in phase 2), the research team discussed the overall findings in a longer meeting and considered that diverse perspectives had emerged, there were no new themes and there was limited variation in the content of new interviews and as such it was considered that the data were saturated. At this point, it was decided to proceed with several interviews to help refine and round out the overall data, prior to phase 3.

### Analysis

Thematic analysis was done iteratively as transcripts emerged and consolidated as a block of analysis following each phase [30]. BOS and KO read the first few transcripts and interview notes and openly coded labels representing the high-level themes, which were informed by deductive reasoning based on the background understanding from the literature. Researchers (BOS, KO, NS) then discussed and agreed on the first set of codes before continuing with further coding using inductive analysis, allowing for the reorganisation of the themes as each subsequent transcript was coded. As coding continued, researchers compared notes and re-read the transcripts to explore the similarities and differences in the data and interpret meaning. Through this process, a more consistent coding framework and set of themes and sub-themes emerged which was rounded at the end of phase 1, explored further to deepen the analysis in phase 2 and refined and confirmed in phase 3 [31]. The analysis was done using word processing software. Reflexivity, thick description, triangulation were supported by the multi-phased methods, cross-referencing between researchers, re-reading the original literature, transcripts and notes and gathering the feedback from the Project Advisory Group [31]. Following phase 3, the research team agreed that sufficient and consistent meaning had been generated from the data across a diversity of perspectives. The research was guided by qualitative research standards [36].

This project had ethical approval from Monash University #44,808, 11 Nov 2024.

## Results

Of 189 expressions of interest, the research team selected 41 participants who joined focus groups and interviews in phases 1 and 2 (Table 2). Of these 10 were in the Project Advisory Group (consulted in phase 1 and 3). Overall, the data encompassed 57 h/1,140 pages. Altogether, 80% of the Project Advisory Group and 68% of wider participants were IMGs, 46% from rural areas and 27% regional. Respondents covered multiple roles 33% had decision-making functions, 20% training teams, 28% supervisor and 18% were IMG trainees. Any quoted material in the results has sub-text indicative of participant characteristics (as indicated in brackets in Table 2) to aid interpretation.

**Table 2:** Participant characteristics ^a^

Characteristics	*N* = 41
*Roles (more than one possible)*	
Project Advisory Group (PAG)	10
Decision makers (SH)	14
Training teams (TD)	15
Supervisors (Sup)	20
IMGs trainee (reg)	13
*Rurality*	
Metropolitan (Met)	11
Regional (Reg)	11
Rural (Rur)	19
*IMG*	
Yes (IMG)	28
No (notIMG)	13

^a^ Participants covered diverse pre-vocational and vocational employment and training pathways for rural general practice training in Australia

Many had multiple roles but the data are displayed according to their main role. Rurality was based on the Modified Monash Model ‘Rur’ – MMM4-7, ‘Reg’ – MMM2-3, ‘Met’ – MMM1 [27]

The thematic organisation evolved through each phase of the research resulting in a main finding of three temporal stages representing different challenges for IMGs including migrating to a new country and acclimatising, moving to new workplaces and communities and training as a specialist rural GP in Australia (Table 3). Two cross-cutting themes were system complexity and frustration and the informal exchange of information and advice from other IMGs (driven by empathy for the challenges incurred). More nuanced sub-themes are summarised (Table 3) and described below.

**Table 3:** Main themes, cross-cutting themes and sub-themes related to IMG challenges

Main themes	Cross-cutting themes	Sub theme
*Migrating to a new country and acclimatising*	▹ Complexity and frustration ▹ Informal exchange of information and advice from other IMGs	• Multiple siloed agencies • Lack of centralised information and tailored real-time advice • Limited information to plan Australian life and career • Limited information about incentives • Self-resilience • Cost of private sector resources • Social and professional isolation
*Moving to new workplaces and communities*		• Finding jobs in isolated towns where locally trained workforce is under-represented • Relocation fatigue • Limited information about rural communities • Further social isolation in rural areas • Informal advice to find supervised employment • Lack of recognition of previous skills and experiences • Limited shadowing options and uncertain about expectations other doctors had of their skills in local context • Practices focused on service rather than learning • Limited contact with domestic cohorts and learners • Variable standards of quality of supervision • Supervisor capacity limited • Compliance based reporting is threatening • Potentially biased reporting • No audit or whistle-blower mechanism where there is inadequate supervision
*Training to become a specialist GP in rural Australia*		• Many pathway options without clear information • Tailored advice about suitable GP training options (not knowing any GPs) • Least training resources and highest need, adding to personal costs • Recurrent changes to training eligibility and structure • Bridging skills and professional practice differences • Bedside manner and consumer-led healthcare • Healthcare team roles and specialty referral options • Medico-legal practice • Wider scope of rural general practice work including diagnostic testing, limited backup, clinical reasoning processes and practising with uncertainty (comfort to ask for help) • Special populations including First Nations • Communication jargon and terminology (and communicating and wiring in English for some) • Different administrative systems, plans and processes • Understanding workplace culture • Getting support for learning from other IMGs

### Migrating to a new country and acclimatising

IMGs found the complexity of migration-related systems both complicated and stressful. Many agencies were perceived to work in siloes: “it’s very hard to understand and to navigate” (30_reg_IMG_Rur). Some IMGs relied on the informal IMG network to find out about key agencies supporting IMGs in rural GP careers: “When talking to other IMGs, I let them know, ‘have you checked the rural network’? And they said [say], ‘what is that?’” (33_reg_IMG_Rur).

IMGs tended to piece together information about migration, employment and education systems from websites: “You just go on internet and you read whatever is available” (39_SH_IMG_Met). Although websites and helplines were not necessarily responsive nor specific enough for answering IMG questions: “I’ve been on AHPRA [registration] line for hours, to Medicare for hours…they said go to that webpage, it explains everything… it wasn’t responding to my questions…” (33_reg_IMG_Rur). Many found tailored advice through informal channels: “I did lots of Googling and there’s like Facebook groups and they tell you what to do…” (40_reg_IMG_Rur).

Information for broader career planning could be hard to find: “pathways are not clear for IMGs …It’s so complicated” (21_reg_IMG_Rur). Further, some IMGs wanted more practical information about living in Australia: “… how set up a bank account or … how Australian politics works or … how Australian social systems work” (32_reg_IMG_Rur). There were signs that IMGs may not know about their eligibility for various rural work incentives: “I lost $12,000 from not getting my … [workforce incentive] WIP payment…. nobody had told me about that” (20_Sup_IMG_Rur).

Some noted that IMGs needed self-reliance: “you give them nothing and expect them to suddenly work it all out themselves…” (15_SH_notIMG_Met). Often IMGs sought costly private sector resources to help: “At the end of the day, some people end up spending up to $15 to 20,000 just trying to get through” (36_reg_IMG_Reg). However, many IMGs experienced frustration, leading to social and professional isolation: “All I wanted was to get into the system …And it was really challenging … it’s just my husband and I…. You feel like an outsider trying to invade that close-knit circle” (36_reg_IMG_Reg).

### Moving to new workplaces and communities

Employment opportunities were complex to navigate and roles were in isolated towns where local workforce was under-represented: “the offers I got as an IMG … was [were] two, three hours in the middle of the desert … So, the job offers was [were] so hard” (33_reg_IMG_Rur). Many IMGs had relocation fatigue: “I’ve moved around enough…I just wanted to settle down…” (40_reg_IMG_Rur). They had limited information about rural places: “I took hours in a day, looking to the map…and in the street walk in the Google map” (33_reg_IMG_Rur). Moving to rural locations involved: “social isolation” (15_SH_notIMG_Met); and “isolation from anything that they know” (12_SH_notIMG_Met).

Job matching for suitably supervised roles could be limited and IMGs may rely on informal advice for employment: “I looked for that on the social media a lot… I searched the whole internet for … a job as an IMG under supervision” (33_reg_IMG_Rur). Previous skills and experiences were not necessarily acknowledged: “She took my resume and just skimmed the first page…. [said] we don’t need you…I ended up in my car just crying …I was used to being someone important” (31_reg_IMG_Rur).

Entering the workplace involved limited opportunities to build confidence: “I had nobody to shadow… they probably expected I was a senior doctor … I had no self-confidence” (30_reg_IMG_Rur). IMGs could also work in settings with limited learning and career progression opportunities: “their [employers] primary interest is in delivering services…not in making them great doctors” (15_SH_notIMG_Met). IMGs expressed similar sentiments: “I felt like I was there to make him [supervisor] money more than anything…” (20_sup_IMG_Rur). Access to other learners and domestic cohorts could be highly variable: “we don’t have any Australian doctor … there’s a different level of learning and work” (34_reg_IMG_Rur).

Supervision quality could be variable: “a lot of them [supervising IMGs] are not accredited supervisors….” (15_SH_notIMG_Met). IMGs confirmed: “I think there is no [supervision] standard” (31_reg_IMG_Reg). Supervisors in rural areas could be heavily stretched: “the capacity to provide that [level 1 supervision] is becoming more difficult…. there’s limited workforce …” (22_TD_notIMG_Met).

The compliance-based reporting required of IMGs once employed, could be threatening: “every three months you’re threatened by a report” (4_PAG_IMG_Rur). There could be a conflict of interest where supervisors authorised IMG performance reports: “her assessment [was] done by the same supervisor who was the bullyer, and that supervisor did a horrible assessment” (31_reg_IMG_Rur). It was noted: “there’s not a whistle-blower… way for people that are vulnerable to be supported at present” (16_SH_notIMG_Rur).

### Training to become a specialist GP in a rural location

IMGs found GP training pathways were complex: “I had no idea which there’s so many pathways to become a GP and it took ages for me to figure out” (30_reg_IMG_Rur). IMGs may not have information about their best training options: “I don’t think they’re educated … as to what’s most appropriate for them” (24_TD_NotIMG _Met). Hospital-based IMGs also had limited access to GPs to inform GP career decisions: “No one in the hospital can tell you anything about it” (40_reg_IMG_Rur).

The eligible training pathways for many IMGs were costly (self-funded, rather than government funded) training options: “we put them in the hardest places to work, unsupported, and they can’t get into the well-funded training programs” (10_PAG_notIMG_Met). This led IMGs to rely on: “spending our strained finances” on additional training resources (7_PAG_IMG_Rur). IMGs also found the security and reliability of rural GP training pathways impacted by frequent changes: “Every two years, it [the program] changes… it’s very confusing… it’s becoming a complicated by day” (21_reg_IMG_Rur).

Some IMGs found the foundational clinical knowledge for medicine was similar in Australia as in their home country. However, there were gaps in information to understand the Australian health system and learn certain skills such as the bedside manner and nuance of consumer led healthcare within Australia: “in my hometown … it’s like more doctor dominated” (31_reg_IMG_Reg); difference in the healthcare team roles and pathways: “allied health and some specialties like rehab and geriatrics … are very rare in other countries” (26_TD_notIMG_Met); and any medico-legal dimensions of rural GP work: “how the Australian medical system functions … the regulations, particularly in the rural areas” (27_reg_IMG_Reg).

The rural scope of general practitioner work was considered the broadest internationally, requiring adapting to new clinical reasoning processes: “the application of their knowledge is…it’s completely different” (15_SH_notIMG_Met); influenced by less access to diagnostic equipment: “we do have x-ray once a week…if anyone has a fall … we need to wait till Tuesdays” (33_reg_IMG_Rur); less back up and referral networks: “you don’t have tertiary hospitals at your fingertips” (39_SH_IMG-Met). Within this context, IMGs sought guidance about practising with uncertainty and accepting mistakes: “[IMGs are] used to not asking for help because that would be seen as incompetence” (26_TD_notIMG_Met). Other rural specific challenges were learning about local cultures and communication styles: “Sometimes it’s a language thing. Sometimes it’s the slang and the local jargon and terminology” (23_TD_notIMG_Met).

Other pragmatic challenges for IMGs were learning the various systems and administrative processes like: “electronic prescribing” (26_TD_notIMG_Met) and the workplace culture: “[they can be] from systems that have a much more rigid hierarchy…addressing your boss by your first name … that’s not allowed” (32_reg_IMG_Rur). The learning demands and lower levels of comfort to ask for help, could lead IMGs to seek: “a lot [of] help from the colleagues [other IMGs] rather than from…official way” (31_reg_IMG_Reg).

## Discussion

This research adds to the wider evidence, a consolidated view of the spectrum of three integrated challenges IMGs experience to navigate their way into a career in rural general practice in the Australian context. At a systems level, the longitudinal perspective lays the foundation for developing more integrated and coordinated strategies to support IMGs towards a GP career in a rural area, as well as zooming into specific issues within the stages. This is preferable over conceptualising IMG issues at a point in time because it showcases the need for more continuous, holistic pathway support, centred on achieving GPs in rural areas. The pathways approach and our results could be further explored and adapted by other countries seeking to understand how to support migrant doctors in particular specialties and contexts.

An integrated view of the pathway challenges suggests that poor upstream support could compound negative feelings and impact training outcomes for IMGs. Wider research highlights IMG issues may follow Maslow’s hierarchy of needs in that foundational needs underpin the achievement of self-actualisation goals [34]. In our study, foundational needs upon arriving in a new country involve finding information about life, work and training systems, as a period of major dislocation and social isolation. In latter stages, IMGs have issues with honing particular skills to work safely and go on to specialise as a GP in rural locations, where they may encounter place-based and professional challenges. At any point in time, IMGs may be moving between these stressors, particularly where issues were unresolved, which could impact work and training outcomes. It might be important for staff and agencies working with IMGs to broaden their holistic support for the IMG experience including promoting their comfort, confidence, belonging and bonding, rather than simply addressing competencies, which has been shown to effectively support rural GPs in other research [25]. Our research findings align with those of other countries where IMGs may have trouble integrating into different health and education systems [20]; face dynamic challenges and experience levels of dissonance when establishing new careers [21].

A major cross-cutting theme was that IMGs rely heavily on informal support from their IMG peers. This places an unfair and unpaid burden of work on an already stretched IMG cohort and risks the circulation of inaccurate or outdated information. It is a public responsibility to improve official information and tailored advice for IMGs. One option is to employ IMGs as paid mentors and supervisors across the pathway [25]. Immediately upon migrating, it might also help if IMGs were routinely given resources to learn about rural general practice careers and pathway requirements so they can seek employment options which can match GP specialty training requirements.

IMG advancement may also rely on more continuous high-quality supervision across the pre-vocational and vocational experience. This could be enabled through more equitable access to bridging programs in rural areas and support for rural general practice supervisors, a group that is stretched due to workforce shortages [19, 37]. Remote supervision, scalable through simple technology and artificial intelligence could add capacity [38]. More publicly led investment could also match IMGs to comprehensive learning environments aligned with IMG registration levels. This could build more security for IMGs to achieve rural GP careers, without the personal cost and commercial interests of private recruiters. There also needs to be more quality oversight of the quality of supervised learning for IMGs with opportunities to safely relocate to new workplaces if encountering unsafe and unfair conditions. The supervisor workforce may also need better resources for orientation, professional development and payment for their role of supervising IMGs. Our findings also identify specific knowledge-based content areas and contextual learning for IMGs entering work and specialising as a GP in a rural area. This can inform what supervisors need to teach across the pathway.

Finally, countries may need to introduce more options for professional networking for IMGs to meet domestically trained cohorts across rural pathways to develop a sense of belonging within the rural GP profession, with some examples of remote supervision and networked professional models for a mostly IMG cohort of rural GP trainees [25, 39].

Our study has limitations. We found consistent and diverse themes by including a breadth of perspectives and IMG insights across rural GP training programs; however, the themes and the findings may require further adaptation to different IMG characteristics, countries of origin and migration, prior qualifications and experience, migration pathway, training models, moratorium requirements and specialties. This research did not include pre-migration challenges which could also have ongoing impacts.

## Conclusion

The challenges of IMGs are complex however, when viewed longitudinally and centred on a country’s goals of achieving specialist GPs working in rural locations, they may provide a basis for planning a set of responsive, comprehensive and coordinated supports. The findings suggested that IMGs could be supported by centralised information systems, tailored support and matching employment in supervised learning. They may also require quality training systems, nuanced to the pressures of learning and development in more isolated rural settings when already experiencing socio-cultural dislocation. This could rely on focused efforts to alleviate IMGs relying on informal peer support to navigate rural GP careers in a new country. Applying these findings to collective strategy could benefit the uptake and retention of skilled IMGs serving rural communities across a broader scope.

## Data Availability

The datasets used and/or analysed during the current study are available from the corresponding author on reasonable request subject to ethical approval and approval of the funding body.

## Abbreviations

AHPRA: Australian Health Practitioner Regulation Agency
AMC: Australian Medical Council
GP: General practitioner
GPSA: General Practice Supervision Australia
IMG: International medical graduate
